# A cage-based training, cognitive testing and enrichment system optimized for rhesus macaques in neuroscience research

**DOI:** 10.3758/s13428-016-0707-3

**Published:** 2016-02-19

**Authors:** A. Calapai, M. Berger, M. Niessing, K. Heisig, R. Brockhausen, S. Treue, A. Gail

**Affiliations:** 1 Cognitive Neuroscience Laboratory, German Primate Center, Kellnerweg 4, 37077 Goettingen, Germany; 2 Bernstein Center for Computational Neuroscience, Goettingen, Germany; 3 Faculty of Biology and Psychology, Goettingen University, Goettingen, Germany

**Keywords:** Cognitive neuroscience, Non-human primates, Automated testing, Animal housing, Animal welfare, Environmental enrichment, Behavioral management

## Abstract

In neurophysiological studies with awake non-human primates (NHP), it is typically necessary to train the animals over a prolonged period of time on a behavioral paradigm before the actual data collection takes place. Rhesus monkeys (*Macaca mulatta*) are the most widely used primate animal models in system neuroscience. Inspired by existing joystick- or touch-screen-based systems designed for a variety of monkey species, we built and successfully employed a stand-alone cage-based training and testing system for rhesus monkeys (e**X**perimental **B**ehavioral **I**ntrument, XBI). The XBI is mobile and easy to handle by both experts and non-experts; animals can work with only minimal physical restraints, yet the ergonomic design successfully encourages stereotypical postures with a consistent positioning of the head relative to the screen. The XBI allows computer-controlled training of the monkeys with a large variety of behavioral tasks and reward protocols typically used in systems and cognitive neuroscience research.

**Electronic supplementary material:**

The online version of this article (doi:10.3758/s13428-016-0707-3) contains supplementary material, which is available to authorized users.

## Introduction

In conventional neurophysiological experimental settings, macaque monkeys normally are required to temporarily leave the housing facility to be trained in dedicated experimental settings outside their cage environment. Animals are therefore moved, by means of a *primate chair,* into a dedicated room or area (here referred to as a *setup*) equipped with the apparatuses needed to run the experiment. In the setup the animals are trained to solve behavioral and cognitive tasks, usually by operating levers, sensors, or touch-screens, while their behavior, for example eye and hand movements, is monitored and, once the training has been completed, their brain activity can be recorded. This classic procedure has been widely used for decades to bring animals to the expertise level required for a given experiment in cognitive neuroscience. However, such a procedure limits the scope of research questions in terms of social and motor behavior, limits self-paced engagement of the animal in the behavioral task, and may give rise to animal welfare concerns due to movement constraints during the sessions in the setup. Overcoming these limitations by providing a cage-based training and testing system opens opportunities to investigate a broader range of activities, such as social behavior, by keeping the animal in its housing environment, together with its social group members (for a review see: Drea, 2006; Fagot & Paleressompoulle, 2009), or motor tasks, by removing body movement constraints (McCluskey & Cullen, 2007). From a training perspective, the potentially more self-paced interaction of the animal with the device, rather than an experimentally imposed training schedule, might create a motivational advantage, with a corresponding learning benefit (Andrews & Rosenblum, 1994; Evans et al., 2008; Gazes et al., 2012; Washburn et al., 1989). From an animal welfare perspective, physical constraints and periods of separation from the peer group in the setup should be refined, reduced, and replaced where possible (3R principle; Russell & Burch, 1959). Even though positive reinforcement training (Fernström et al., 2009; Perlman et al., 2012; Schapiro et al., 2003) is routinely used in neuroscience research to accustom animals to physical movement restraints step-by-step over extended periods, one cannot fully rule out a detrimental effect of movement restraints and setup isolation on well-being. Even for experiments that require physical constraints for scientific reasons, there can be early phases of behavioral training where movement restraints are not yet necessary. Such testing and training therefore could be conducted in the animal’s housing environment, perhaps even while maintaining the monkey’s social situation.

With the XBI (e**X**perimental **B**ehavioral **I**ntrument) we developed a cage-based, yet mobile and remotely controllable behavioral testing system for rhesus macaques in research-typical housing environments (for similar devices see Andrews & Rosenblum, 1994; Fagot & Bonté, 2010; Fagot & Paleressompoulle, 2009; Gazes et al., 2012; Mandell & Sackett, 2008; Rumbaugh, Hopkins, Washburn, & Savage-Rumbaugh, 1989; Richardson et al., 1990; Truppa et al., 2010; Washburn et al., 1989; Washburn & Rumbaugh, 1992; Weed et al., 1999). To minimize management requirements, the system is very robust and spray-water resistant. For maximal comparability, the XBI mimics conventional neuroscience settings in that it uses a precise fluid reward system. Also, the view of the visual display and physical access to the touch-screen is only minimally constrained, as is desirable for most cognitive neuroscience studies, while maintaining a uniform screen-eye distance. Finally, to allow behavioral assessment beyond the immediate task performance as registered by the touch screen, e.g., analyzing facial expressions of the animal, the XBI includes video surveillance with a full-body frontal view of the animals during task performance.

Here, we provide a technical description of the XBI and preliminary behavioral tests as proof-of-concept, including data on the initial experiences of naïve animals with the XBI. We also provide an account of our experience with the device in the daily routines of an animal housing facility.

## Methods

The XBI is designed as a device for training and behavioral testing of rhesus macaques in their housing environment, and can also be used for environmental enrichment. It has been developed with five design requirements in mind. First, the device needs to be cage-mountable to allow easy access for the animals without human interference (Gazes et al., 2012; Richardson et al., 1990; Truppa et al., 2010; Weed et al., 1999) or having to restrain the animals during transportation to the setup. Second, the electronics and other internal parts need to be protected against dirt and spray water typically present in such environments. Third, the XBI must be robust to resist potential forces applied by the animals. Fourth, operating the device should be easy enough to be handled by different people, including non-scientific personnel. Finally, the XBI’s hard- and software should be flexible enough to allow for a wide variety of training procedures and experimental task designs. This includes complex visually instructed cognitive tasks with well-defined stimulus viewing conditions and a high degree of flexibility in how the animal interacts with the device.

**Fig. 1:**
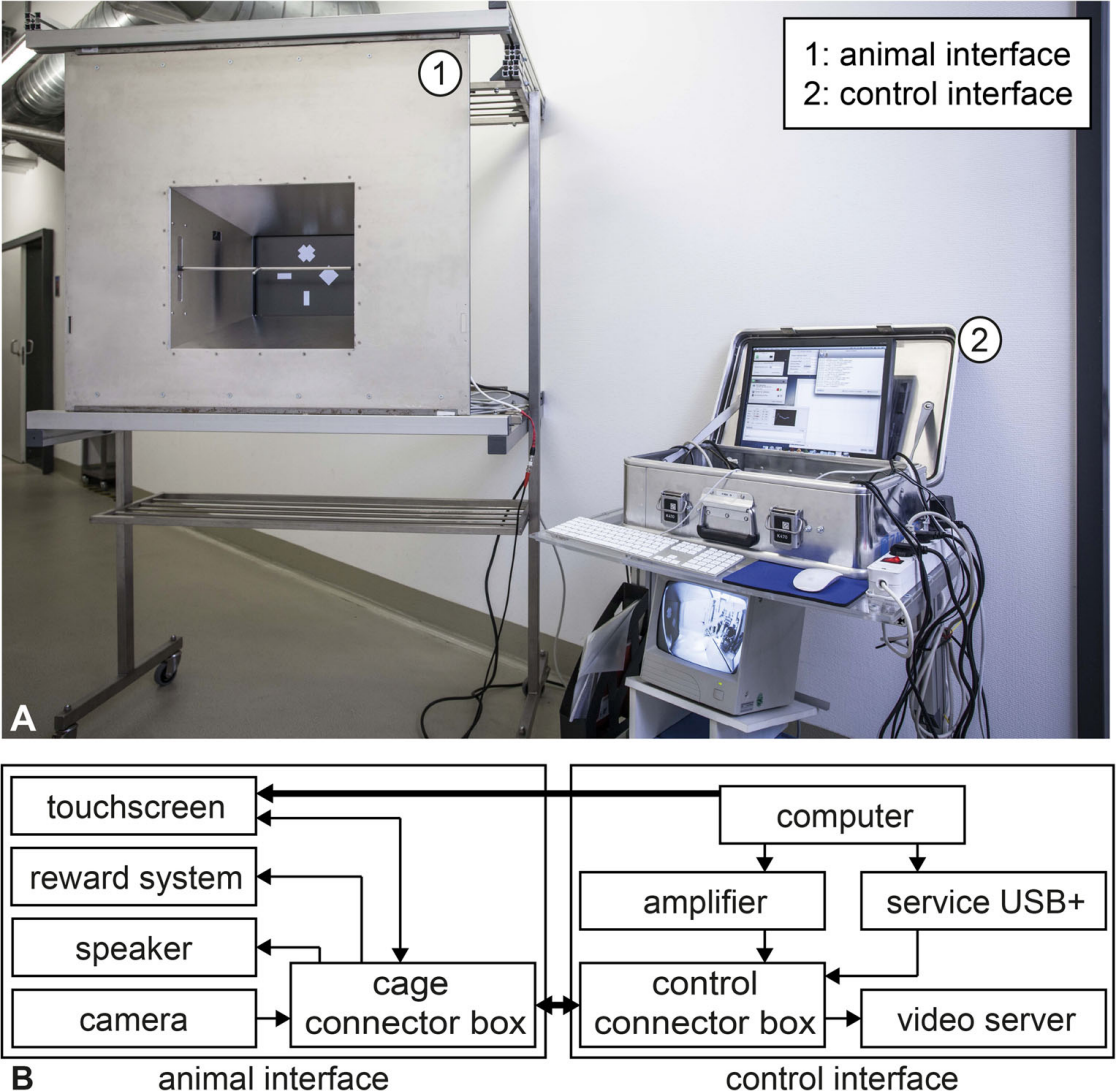
**A** Image of the XBI. (1) Animal interface (AI) in the wheeled frame. A modified version of this frame is used to mount the AI on the front compartment in cases where it could not be anchored directly. (2) Control interface (CI) on a custom-made cart designed for easy relocation and accessibility. **B** Schematics of the XBI. Thick arrows represent connections between the two interfaces and thin arrows represent internal connections between elements of the same interface. The direction of an arrow represents the direction of the signal

To address these needs the XBI’s hardware is divided into two parts: the animal Interface (AI) and the control interface (CI) (Fig. 1). In the following, we will describe the main design features and technical specifications. More detailed information on custom-built parts or purchased equipment are available upon request from the corresponding author.

### Animal interface (AI)

**Fig. 2:**
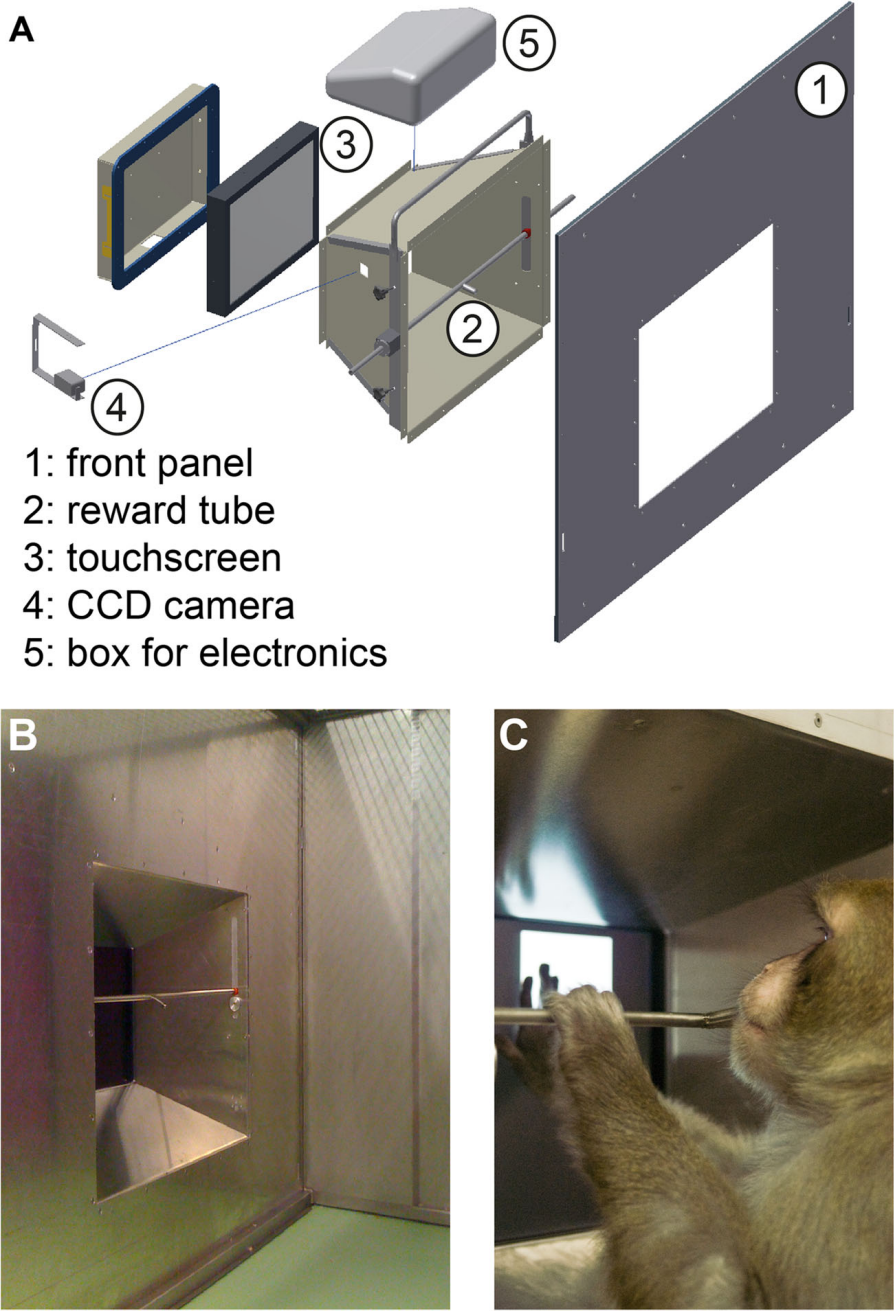
**A** Exploded-view drawing of the XBI’s front, facing the animal. From left to right: the protective frame for the touch-screen, the touch-screen, the funnel, and the reward tube, the mounting frame for cage anchoring. **B** XBI front from the animals’ perspective. **C** One animal working at the XBI, in a trial of the touch-hold-release task

The AI, used inside the animal facility, is the part of the XBI to which the animal has access (Fig. 2). It consists of mechanical and electronic components. For handling and safety reasons, the mechanical parts are lightweight and, where possible, built from aluminum. The dimensions of the whole device are 106 cm × 93 cm × 30 cm (W × H × D) and it weighs approximately 23 kg. By reducing the size of the outer frame and using lighter panels, we expect to substantially reduce the weight of future versions. The AI can be stored or transported using a custom-built wheeled frame (Fig. 1A), providing comfortable access to the front and rear for cleaning and maintenance. The XBI can be used either with the cart (no lifting required) or by directly attaching it to the animal’s enclosure (freeing the cart). For safety reasons all electronics of the AI run on low-voltage (maximum 12 V). Parts close to the animal that have to be powered include the touch-screen as the interaction device, a peristaltic pump for delivering reward, a loudspeaker to provide feedback or instructions, a surveillance camera for remote observation, and a cable connector box to minimize the number of cables between both interfaces. The rest of the XBI electronics reside remotely in the CI.

All animals had access to the AI in their home enclosures. These consisted of a room-sized group compartment and a smaller front compartment, physically separable by a dividing gate. The AI is attached to the front compartment with an aluminum-mounting frame, replacing one side panel of the compartment (Fig. 2B). For nine out of 11 animals the front compartment was connected to the group compartment such that the tested animal could be seated on-sight with peer animals. For two out of 11 animals the arrangement of the front compartment with respect to the group compartment did not allow visual contact.

The middle part of the XBI-AI is shaped as a funnel that narrows to the dimensions of a touch-screen (ELO 1537L), such that only the 15-in. LCD display is accessible for the animal. The dimensions of the front opening of the funnel are 48.6 cm × 41 cm (W × H) and the distance to the screen is 26.2 cm. This distance was chosen based on prior experience with rhesus macaques interacting with a touch-screen in neurophysiology experiments in our laboratory (Gail et al., 2009; Westendorff et al., 2010). The display is operated at a resolution of 1024 × 768 at 75 Hz. The touch panel in front of the display utilizes ultrasonic waves in combination with piezoelectric transducers for the sensing of the touch signal with a positional accuracy of 2.5 mm or better. The touch-screen is designed to be resistant against mechanical forces. A stainless steel tube with 8-mm inner and 12-mm outer diameter reaches across the funnel, at a fixed distance of 24 cm from the touch-screen. Fluid reward is delivered through a 1-mm opening in a 30-mm spout in the middle of this tube, precisely controlled via a peristaltic pump (see below). The stainless steel tube with the spout can be rotated and adjusted horizontally and vertically in position. In this way it is possible to set it to comfortable positions for individual monkeys of different size. Given that the animals usually operate the device with the reward tube as close as possible to their mouths (Fig. S1), the eye-to-screen distance is around 28–32 cm, depending on an individual’s head orientation and size. The screen size of 30.4 cm horizontal and 22.8 cm vertical provides 54° of visual angle along the horizontal and 42° along the vertical axis.

The AI’s backside contains a reward unit consisting of a fluid container (2.5-L plastic bottle), connected to the metal reward tube using flexible PVC tubes with 6-mm inner diameter. These tubes are exchanged after every 2 weeks of use. A peristaltic pump (Verderflex OEM M025 DC) allows electronic control of the reward flow. This reward unit can be placed at either the left or right outer side of the funnel to adapt to different cage structures. The pump delivers 1.8 ml/s of activation time, with a precision of approximately 0.01 ml. The reward was precisely timed and dosed via the experimental control software, which is crucial for cognitive neuroscience testing.

A mono sound transducer (Visaton, SpeaKa 130 mm) is glued on the outside of one of the funnel walls, using the wall as resonator for sound amplification. A compact 160° wide-angle CCD camera (ABUS TV7512) with 480 TV lines (438 kPixel) resolution is attached to a small opening in the metal funnel, protected by a clear polycarbonate window. The wide-angle view enables monitoring of the monkey and of the video screen at the same time.

Except for the VGA video cable, all connections (including power and signal lines) are routed to the CI via a custom-made connector box and a standard parallel D-SUB 25 connector cable (up to 15 m). Thus, only these two cables have to be routed to the outside of the animal facility. Within the connector cable we used multiple leads for power and ground lines to increase the amount of current that can be delivered through the cable.

The overall maximal nominal power consumption for the AI is 37.6 W (touch-screen 22 W, camera 0.6 W, active peristaltic pump 15 W). With an operating DC voltage of 12 V the XBI draws a maximum nominal current of 3.13 A. In practice we measured a total current of 1.5 A.

The AI is build to be operated for years, even in a dirty and humid work environment such as an animal facility. The front side facing the monkey cage is resistant against feces, urine and direct water impact during cage cleaning procedures. On the backside of the AI all components are protected against spray water and particles larger than 2.5 mm. According to IEC 60529, the international protection marking level of the whole XBI is IP 33, with a substantially higher protection from the inside of the monkey cage.

### Control interface

The CI consists of all the hardware and software needed for controlling the AI. It usually operates from outside the animal facility, weighs 12.2 kg and fits into a transportable box (W: 59 cm, H: 12 cm, D: 38 cm) for easy transport. The CI receives and sends signals from the AI through the VGA and connector cables. A second custom-made connector box distributes all connections from the connector cable to the individual components. The VGA cable as well as the serial RS232 connection from the touch-screen is connected to a computer that controls the XBI (Fig. 1). To control various devices from the computer, we integrated a USB interface (Service USB plus, Böning und Kallenbach). This platform provides multiple analogue and digital GPIOs (General Purpose Inputs/Outputs) which can deliver currents of up to 1.3 A. One of the digital outputs is used for operating the peristaltic pump, while the others have not been used in the context of the experiments described here. In addition, the computer’s audio output is connected to a custom-built sound amplifier, which provides the audio signal for the sound transducer. The camera signal is routed to a video server (TRENDnet TV-VS1P) and from the video server to an analogue screen for on-site observation. The video server and the XBI computer are connected to the Local Area Network (LAN). In this way any computer on the LAN can be used for remotely controlling the XBI as well as recording videos and downloading data.

As long as the necessary interfaces are available, hardware requirements for the CI computer to run the XBI do not exceed those of standard desktop or laptop computers. We used VGA and USB connections with a RS232 adapter for the touch-screen in the AI, another USB port for the Service USB plus device, DVI-D for the CI’s screen, and the headphone audio out for the audio amplifier. Although LAN connectivity is not necessary for the XBI to operate, it provides useful remote control capability. The video server is not directly connected to the computer but can be accessed via LAN. For the computer we either used an Apple Mac mini (2.5 GHz Intel i5, 8 GB RAM) or an Apple MacBook (2.4 GHz Intel Core 2 Duo, 2 GB RAM). The Mac OS is used since it interfaces optimally with MWorks (http://mworks-project.org/). This open-source software is a highly flexible C^++^-based package for designing and real-time controlling behavioral tasks for neurophysiological and psychophysical experiments. MWorks can be expanded by dedicated software plug-ins to serve a wide range of experimental needs. Behavioral tasks are coded as XML files. A custom-made XML editor makes programming and modifying task files easy even for users without programming experience. MWorks runs in a client-server structure. The XBI can be run either as a standalone system or be operated via LAN. Data files are generated on the CI-computer that runs the server software.

### Animals, grouping and fluid control

Overall, a total of 11 male rhesus monkeys (*Macaca mulatta*) were trained on the XBI within their housing facility. Three animals (Gro, Chi, and Zep) had access to the XBI as a group directly from the group compartment of their home cage. We report their behavioral data as group performance. We confirmed that an off-line analysis of the video footage allows for determining which animal was responsible for each of the XBI interactions. Since performance comparisons between individual animals are not the purpose of this report and since future ID tagging will render manual performance assignment to individuals unnecessary, we did not extend our pilot off-line analysis to the full data set.

The other eight animals had individual access to the XBI from within the smaller front compartment of their home enclosures. These eight animals were physically separated from their social group by a dividing wall separating the front compartment from the group compartment during the XBI sessions. Animals Fla, Alw, Nor, Odo, and Pru were in sight with their social group, while animals Han, Toa, and Zor were in sight only with members of other groups in the housing facility.

Most of the 11 animals had at least 2 h of unlimited access to water and fruits before and after each XBI session (Monday to Friday) and 24 h on all other days (see Table 1 for details). Two animals (Pru and Zor) were trained on the XBI under fluid control, in which the XBI provided the only access to fluid on working days (Monday to Friday). Animal Pru, in the early phases of the training, received plain water as reward. The other animals were rewarded with fruit-flavored sweetened water (active O2, Adelholzener) diluted with plain water at a ratio of 1:3.

**Table 1:** For each of the 11 animals (rows) that took part in the two experiments the table lists the fluid access scheme (before and/or after the XBI session), which, if any, of the social group members was undergoing XBI training, which experiment or experiments were used, and the animals’ age at the time of their first encounter with the device

Animal	Fluid access	XBI mates	Experiment	Age (years)
Alw	Before/After	-	AS	4
Chi	Before/After	Gro, Zep	AS	4
Fla	Before/After	-	AS	3
Gro	Before/After	Chi, Zep	AS	4
Han	Before/After	-	AS	3
Nor	Before/After	-	AS	3
Odo	Before/After	-	AS	7
Pru	XBI only, Before/After	Zor	FTS, THR, MS	7
Toa	After	-	AS	3
Zep	Before/After	Chi, Gro	AS	4
Zor	XBI only	Pru	THR	12

*AS* accommodation study, *FTS* free-task selection, *THR* touch-hold-release task, *MS* delayed match-to-sample

Note that monkey Zor, a 12-year-old animal, was tested only during the development phase of the device.

### Behavioral paradigms

To date four units of the XBI are in ongoing use and have been tested in various experiments. All experiments complied with institutional guidelines on Animal Care and Use of the German Primate Center and with European (Directive 2010/63/EU) and German national law and regulations, and were approved by regional authorities where necessary. Two experimental paradigms shall serve as examples of the functionality of the system and acceptance by the animals. The first paradigm, the *accommodation study*, probed the ability of naïve animals to autonomously learn how to successfully operate a touch-screen on a basic level with no formal training (e.g., training to human handling). The second experiment, the *free-task selection* tested the XBI as a cognitive testing system and as an enrichment tool.

#### Accommodation study

Nine animals (age: 4–7 years) participated in the accommodation study (AS). They were naïve with respect to the XBI, and the accommodation study marked their first encounter with the device. Each animal had 90 min of daily access (typically from Monday to Friday) to the XBI over a period of 2 weeks excluding the weekend. None of the animals had previously participated in any type of cognitive training.

In the accommodation study the monkeys had to perform a simple touch task. At the beginning of each trial a steady blue (white for monkey Fla) square target stimulus 20 × 20 cm^2^, was displayed on the screen on a black background. Touching the target for at least 100 ms triggered a fluid reward (successful trial). Touching the background terminated the trial without a reward (unsuccessful trial). Each trial was followed by an inter-trial interval during which the screen remained black. After 1 s without touching the screen the next trial started. This requirement of releasing the touch of the screen prevented the animals from successfully completing a series of tasks by simply keeping a finger (or any other body parts) on the screen. In addition to the delivery of the fluid reward, two different sounds indicated whether a trial was a success or not.

#### Free-task selection

One animal (Pru, 7 years old) participated in the *Free-Task Selection* (FTS). Note that before entering the free-task selection, the monkey underwent 4 months of positive reinforcement training to enter and exit the primate chair and 12 months of training on the XBI (see below for details).

**Fig. 3:**
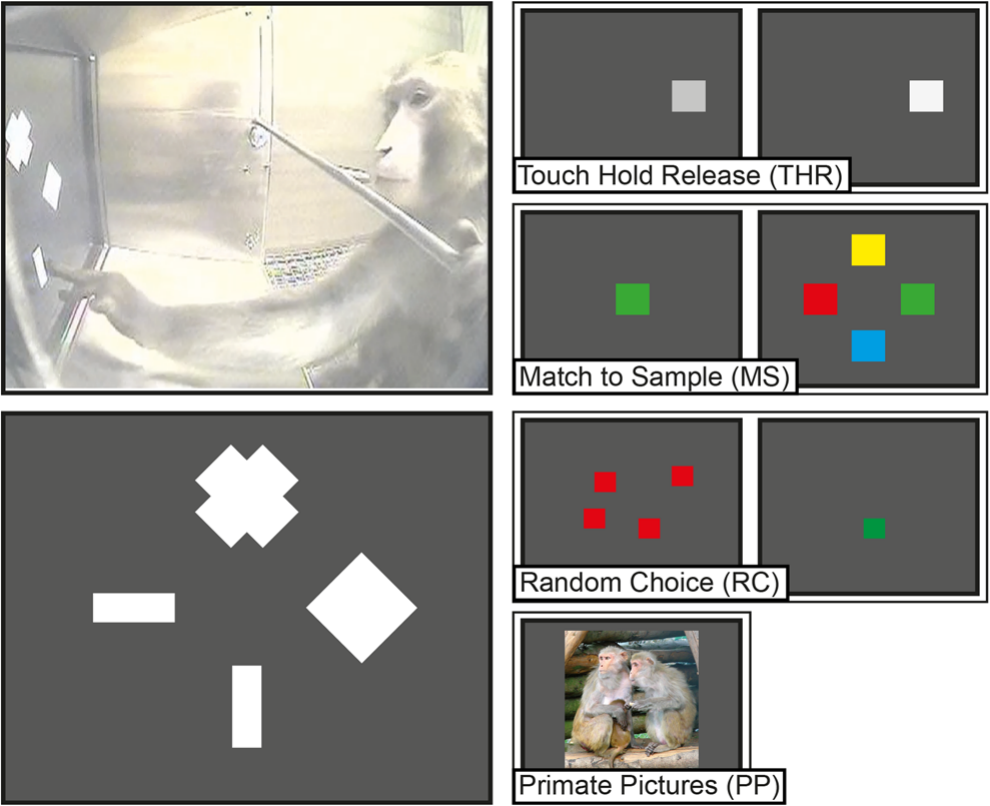
**Left column, top**: view of the internal XBI camera while animal Pru chooses which task to execute next. **Bottom**: representation of the first frame of each trial of the four-choices tasks. Each white symbol is associated with one of the four tasks depicted in the right column, from top to bottom: cross for Touch Hold Release (THR), rhombus for Match to Sample (MS), vertical bar for Random Choice (RC), horizontal bar for Picture Presentation (PP, representative picture)

In the free-task selection, at the beginning of each trial, four symbols were displayed on the screen (see Fig. 3), each one permanently associated with one subtask (Washburn et al., 1991):

- *The cross* was associated with a simple *touch-hold-release* (THR) task, an extension of the touch task in the accommodation study. After the animal selected the cross symbol and after a 500-ms delay the four symbols were replaced by a gray square (5 × 5 cm). The animal had 4,000 ms to reach for the target, which once touched, it brightened. After 500–2,500 ms of maintaining the touch the square dimmed. Now the animal had to release the touch within 500 ms to successfully complete the trial. The position of the stimulus on the screen and the required hold-time were randomized trial-by-trial. For this subtask the average duration of a successful trial was 4.8 s from when the animal selected the cross symbol.
- *The rhombus* was associated with a color-based *delayed match-to-sample* (MS) task. In MS trials the animal had to first touch a colored square (8 × 8 cm) at the center of the screen and after a randomized delay (1.5–3 s), touch the square with the same color amongst four differently colored squares of the same size displayed left, right, above, and below the screen center. The colors of the squares were randomly assigned trial-by-trial. The animal had to select the target within 4 s for correct performance, otherwise the trial would terminate without a reward. The same outcome would occur if the wrong stimulus was selected. For this subtask, the average length of a successful trial was 2.7 s.
- *The horizontal bar* was associated with a *random choice* (RC) task in which the animal had to touch one of four identical 3 × 3 cm red squares that were randomly positioned on the screen. Only one randomly determined stimulus would trigger a reward. By setting the amount of reward to four times the reward in the touch-hold-release and match-to-sample tasks the average reward was equated across these task types. For this subtask the average length of a successful trial was 3.6 s.
- *The vertical bar* was associated with a *primate picture* (PP) task in which one out of 20 photographs of non-human primates were shown on the screen for 5 s. After selection, no additional touch was necessary and no fluid reward was given in this task. For this subtask the average length of a trial was 5.6 s.

The animal was trained on the touch-hold-release task for over 6 months while technical aspects of the XBI prototype were under development and the match-to-sample task for 3 months. Once the monkey had reached a consistent performance above 80 % over 10 sessions (2 weeks) in these two tasks he was introduced to the free-task selection task. It included the two known tasks and the two novel tasks each associated with its corresponding symbol (see above). To determine the influence of relative reward amounts on relative choice probabilities, the first 31 sessions (3 months) of the free-task selection have been collected in two experimental conditions: lower reward RC task (20 sessions) versus higher reward RC task (11 sessions). We statistically verified the influence of relative reward amount on relative choice probabilities by the mean of the Multinomial Logit Model with estimated p-values using pairs cluster bootstrapped t-statistics (Cameron, Gelbach, & Miller, 2008).

## Results

The XBI is designed for behavioral training, cognitive testing, and enrichment of physically unrestrained rhesus monkeys in an animal facility. Both of its components (the AI and the CI) are safely useable for the experimenter and the monkeys in this environment. Below, we will describe the usability of the XBI from the experimenter’s perspective as well as behavioral example data recorded with the XBI as a proof-of-concept for cognitive testing and environmental enrichment.

### Handling by the experimenters

A single person can handle the XBI safely. The use of a wheeled frame for storage and transport allows the XBI to be directly transferred to the sides of a cage avoiding the need to lift the AI. The mesh grid of the cage can be conveniently removed after the XBI has been mounted in front of it.

The XBI can be set up quickly. Given some experience, aligning the device to the cage and preparing a given experiment takes less than 10 min. In this time: the device is mounted to the cage replacing one of the cage’s walls, is connected to permanently installed cables for the electronic communication between the two interfaces, the reward system is filled up, and the task and the video recording are initiated. From this point on the system is able to run autonomously, and without supervision, until it is manually stopped. If needed, the touch-screen as well as the cage are briefly cleaned before starting a new XBI session. This takes less than 10 min. To prevent technical malfunction by accumulating dirt the AI is thoroughly cleaned after about five sessions and the plastic tubes for reward delivery are replaced when needed.

The XBI is robust enough to endure repeated mounting and dismounting. In our setting one of the devices was used daily in three different rooms. Despite the substantial amount of mechanical stress of changing the location of the device multiple times per day over many months, malfunctions that delayed the starting procedure or prevented the system from running altogether were very rare. Most of these malfunctions resulted from cables not properly connected or partially damaged by the frequent use. Switching to more resistant cables eliminated such problems. Other technical issues were not observed. Across four separate XBI devices operated for more than 1 year, only one bent reward tube and one broken peristaltic pump had to be exchanged.

The XBI requires little regular maintenance. The electronic devices attached to the AI are protected against spray water and dirt by their encapsulation. However, water and dirt on the touch surface can interfere with the assessment of behavioral performance by creating false triggers. To reduce dirt accumulation, the floor of the cage in which the XBI was placed was either a mesh or covered with dry wood-chip bedding. Accordingly, regular maintenance is inexpensive in terms of parts and materials. For hygienic reasons, we replaced the silicon tube (1 m) of the reward system after 2 weeks of use.

The XBI is easy to handle. Daily setup routines were performed not only by the experimenters, but also by students and technical assistants. It required only 2–3 sessions under supervision until a person was experienced enough to independently operate the XBI.

The XBI approach is scalable to a larger number of devices. Given the remote control and video surveillance options, we were able to simultaneously control our three XBI devices, even when they were located in different buildings. This allowed one single experimenter to remotely manage the training of several animals.

### Monkey interactions

In the following section we will report behavioral data collected to probe (1) the XBI’s attractiveness to naïve animals and (2) its suitability for cognitive tests.

#### Accommodation experiment: Unsupervised training of naïve animals in minimally restrained conditions

With the accommodation experiment we determined that naïve animals learn to operate the XBI without human instruction, supervision, or intervention. The animals were naïve in the sense that while they had received positive reinforcement training for their handling in the housing environment (moving into and out of the front compartment, holding still, etc.), they had never experienced a touch-screen before and never had been part of experimental procedures or computer-controlled training in a cognitive task. During each of the ten sessions of the accommodation experiment, the animal had the opportunity to freely explore the device. Presumably driven by both their curiosity and the odor of the fruit-flavored water at the tip of the reward spout, eight out of nine monkeys approached first the reward tube and subsequently the shiny aluminum frame of the XBI. For eight out of nine animals, the first successful interaction with the touch-screen occurred during the very first 20 min.

**Fig. 4:**
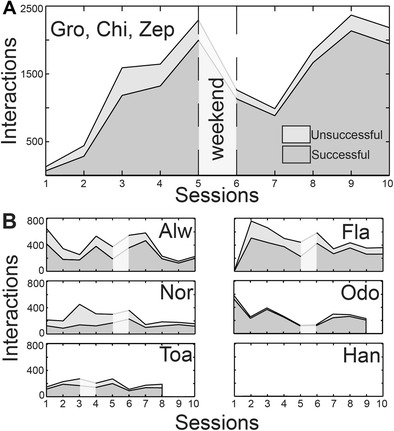
**A** Number of interactions with the XBI system pooled across the monkeys Zep, Gro, and Chi. Successful trials (dark gray area), unsuccessful trials (light gray area), and total trials (top line) are plotted for up to 10 consecutive working days during the first 2 weeks, interrupted by 2 days off (weekend) between the fifth and sixth sessions. **B** Interactions for monkeys Alw, Fla, Nor, Odo, Toa, and Han. Note that animals Odo and Toa underwent respectively nine sessions (for technical reasons) and eight sessions (for unrelated reasons). Animal Toa started his first week on a Wednesday and the break lasted a whole week instead of a weekend. Animal Han did not interact with the XBI’s touchscreen at all during these sessions

During XBI sessions most of the animals were in the front compartment by themselves (with visual contact to their social group, see Methods), except for three (Chi, Zep, and Gro) that had access to the XBI as a group. As shown in Fig. 4A, animals Chi, Zep, and Gro, after gaining some experience with the touch-screen in the first two sessions, substantially increased both their number of interactions with the XBI and the proportion of successful trials in the following days. Although with high variability and different success proportions, animals Alw, Fla, Nor, and Odo showed a substantial interest in the XBI, generating hundreds of successful trials each day and progressively improving their ability to trigger a successful trial (Fig. 4B). Only animal Han showed no interest in the XBI.

#### Free-task selection experiment

The choice proportions of monkey Pru across the four tasks stabilized within the first two sessions. To determine the influence of relative reward amounts on relative choice probabilities, the reward associated with a successful random choice trial was set to three times the reward associated with the touch-hold-release (THR) and the match-to-sample (MS) tasks (PP did not deliver a fluid reward). For the next 11 sessions it was increased to four times.

**Fig. 5:**
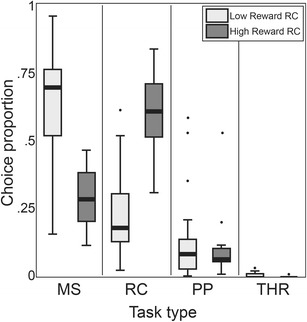
Box-and-whisker plot of the distribution of choices of task type during the free-task selection, in two conditions for monkey Pru. White boxes represent the experimental condition (20 sessions) in which the reward in the random choice task (RC) was three times the amount of reward in the match-to-sample (MS) and touch-hold-release (THR) tasks. Gray boxes represent the experimental condition (11 sessions) in which the RC reward was increased to four times the amount in the MS and THR tasks. The distribution of the difference between higher reward and lower reward was estimated for each task and compared with the other tasks. To achieve such comparison the data set was repeatedly re-sampled by cluster; a model estimated and inferences were made on the sampling distribution of the pivotal (t) statistic. For each comparison the confidence interval for the significance level was weighted by the number of comparisons (confidence interval’s significance level: 1–(0.005/6)) and the confidence interval for each task comparison was determined (MS to RC 0.0566–0.8195; MS to PP 0.3595–3.9314, MS to THR 0.3760–14.7022; RC to PP 1.2448–24.4346; RC to THR 0.8994–132.1582; PP to THR 0.5127–7.6293). P-values for the six comparisons, corrected with the Bonferroni method for multiple testing, are: MS to RC 0.012; MS to PP 1.00; MS to THR 0.78; RC to PP 0.036; RC to THR 0.60; PP to THR 1.00

We statistically verified the influence of relative reward amount on relative choice probabilities (see Methods and Fig. 5 legend for details). We found that MS to RC is the only comparison that yields moderate evidences for a statistical difference (*p* = 0.012), while RC to THR comparison shows a trend (*p* = 0.036) and all the other comparisons show no significant influence by the relative reward amount. This suggests that when the RC task was highly rewarded, the animal selected the RC task more often, at the expense of the MS and THR tasks but not the PP task. As can be seen in Fig. 5, the distribution of MS and RC choice proportion are reversed in the two conditions; the distribution of the THR choices, already very low in the low reward condition, approach zero, while the frequency of PP choices is unaffected. This demonstrates that the fluid reward amounts in the XBI can be used to flexibly and precisely change the animal’s preferences as needed, for example, in decision-making experiments.

## Discussion

We developed the XBI as a cage-based stand-alone device for behavioral training and cognitive testing of rhesus macaques and designed for a seamless integration into conventional neuroscience experiments. We tested the XBI for over a year and found it robust and flexible enough for use in different animal facilities. It is easy to handle such that one non-expert person is able to operate it on a daily basis with short setup times and without the need to remove it during wet cage cleaning procedures. Animals do not have to leave their housing environment and naïve animals learn to interact with the device in an unsupervised fashion, at a self-paced rate within the time window of device access. As a proof of concept, we presented training examples matching neuroscience research questions, e.g., training visually instructed goal-directed movements, but a much broader spectrum of behavioral testing is possible. Despite lacking physical constraints, the animals adopted stereotyped postures, adapted to the ergonomic design of the XBI, creating a well-defined perspective and distance from the visual stimuli and the reach goals on the monitor. The close-up full-body video surveillance embedded in the system allows further behavioral assessments.

Devices similar to the XBI have proved to be highly useful in cognitive assessments of non-human primates (Andrews & Rosenblum, 1994; Fagot & Bonté, 2010; Fagot & Paleressompoulle, 2009; Fagot & Parron, 2010; Gazes et al., 2012; Mandell & Sackett, 2008; Rumbaugh et al., 1989; Richardson et al., 1990; Truppa et al., 2010; Washburn et al., 1989; Weed et al., 1999). In systems and cognitive neuroscience research additional features of such devices are desirable, which we implemented to increase the range of possible uses for the XBI.

First, most existing systems use solid rewards (Andrews & Rosenblum, 1994; Fagot & Bonté, 2010; Gazes et al., 2012; Truppa et al., 2010; Weed et al., 1999), with the exception of Mandell and Sackett (2008). We use fluid rewards for the XBI, since in typical neuroscience behavioral protocols, rewards need to be precisely dosed and timed, e.g., for decision-making studies with fine-grained reward schedules (for example: Klaes et al., 2011; Platt, 2002; Sugrue et al., 2004) and as reinforcers in eye-position contingent, complex visual, and sensorimotor tasks (for example: Gail et al., 2000; Gail & Andersen, 2006; Katzner et al., 2009; Niebergall et al., 2011; Patzwahl & Treue, 2009).

Second, to be suited for a large range of neuroscience questions, the monitor and interactive touch surface should be easily accessible. In most of the touch-screen-based systems using radio-frequency identification (RFID) the monkeys need to reach through ports equipped with antenna coils, to reliably read the RFID tags (Andrews & Rosenblum, 1994; Fagot & Bonté, 2010; Gazes et al., 2012). We do not use view and reach ports to not constrain reaching movements toward and across the touch-screen and because preliminary technical tests indicate that our design is suitable for hand-specific RFID tagging without such ports. A further advantage of not having ports or physical shielding of the touch-screen is the unobstructed full-body frontal video image of the animal in the XBI, which can be used for various forms of behavioral assessments, e.g., more complex video-based motion tracking, analysis of emotional facial expressions, etc. On the other hand, we want to encourage an ergonomic posture of the animals with a defined viewing distance from the screen. In systems without reach or view ports the screen was placed in the same plane or close to the wall of the cage, allowing the animals more freedom in choice of the posture and screen-eye distance (Gazes et al., 2012; Truppa et al., 2010; Weed et al., 1999). Since many studies in the neurosciences use visually guided tasks, it is critical to provide a controlled visual stimulus, including a well-defined retinal size. We achieved this by positioning the reward tube and touch-screen at opposite ends of a funnel, with the funnel depth adjusted to the arm lengths of rhesus monkeys and the reward tube position optimized for their sitting posture. With the aid of the integrated full-body video recordings, we verified that the animals quickly adopted a desirable and stereotypical posture in front of the screen, with the face in front of the screen and the mouth at the opening of the reward tube (see Supplementary Fig. 1 and supplementary videos). In future, this will presumably allow for an easy integration of video-based eye-tracking and face-recognition systems. Moreover, given the central placement of the reward spout, animals were free to use either hand for interacting with the device (see monkey Nor and Fla in Supplementary Fig. S1 and video).

Third, we designed the XBI to be compact and mobile, including remote control via LAN (Mandell & Sackett, 2008, 2009). This makes individual devices easily transferable between rooms, floors, or even buildings, and adaptable to different enclosures. Using one server we simultaneously operated our three devices in two buildings, switching them amongst six social groups.

Finally, we believe that the spontaneous and continued engagement of the naïve animals that we observed during early exposure to the XBI, despite no restrictions on fluid intake, shows that cage-based devices, beyond showing great potential as an alternative to some conventional setup training for neuroscience research, can also serve as valuable tools for environmental enrichment, in compliance with the 3Rs principle (Evans et al., 2008; Fagot et al., 2014; Richardson et al., 1990; Russell & Burch, 1959; Washburn et al., 1991; Washburn & Rumbaugh, 1992). It is important to note that the XBI does not trigger the same level of interest in all naïve animals (Evans et al., 2008). We are currently expanding these observations in a separate study to address the need for more systematic behavioral profiling of such inter-individual differences.

## Electronic supplementary material

Below is the link to the electronic supplementary material.

ESM 1 (DOCX 2130 kb)

ESM 2 (MP4 9.59 mb)
