# Supplementary material for: A cage-based training, cognitive testing and enrichment system optimized for rhesus macaques in neuroscience research
Source: Behav Res Methods. 2016 Feb 19;49(1):35–45. doi: 10.3758/s13428-016-0707-3 (PMC5352800; doi:10.3758/s13428-016-0707-3)
Supplement: Supplementary file 1 — (DOCX 2130 kb) [file 13428_2016_707_MOESM1_ESM.docx]

**Supplementary Material**


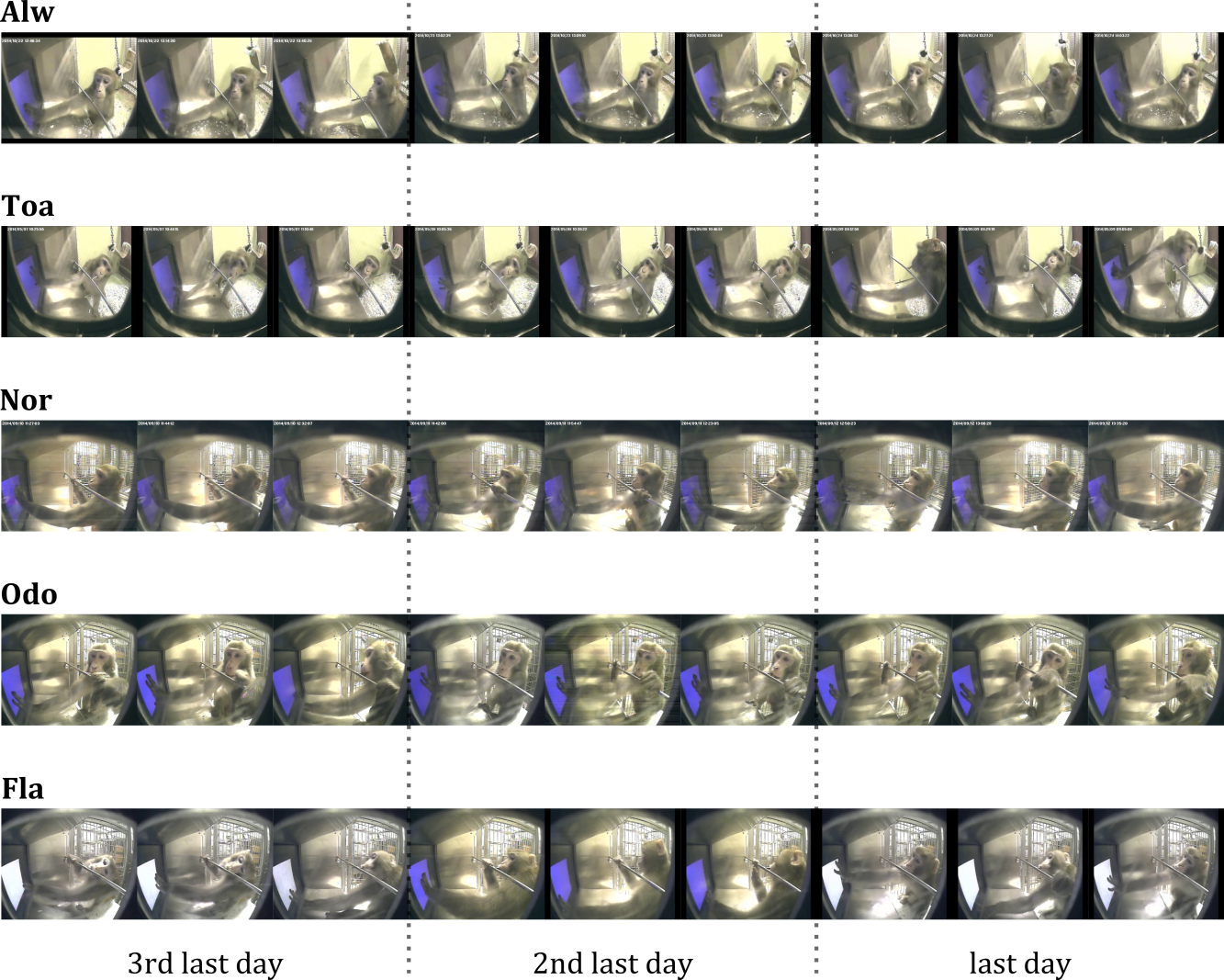


To test if each animal operated the XBI in a stereotypical way with a consistent posture, we analyzed the last three days of the accommodation experiment (section 4.2.1) for animals Alw, Toa, Nor, Odo and Fla. Animals participating in the accommodation study were introduced to the XBI for the first time on the first day of the experiment and did not undergo cognitive training before. Posture data was collected as follows: For each of the last three accommodation days and for each animal we chose three times three correct interactions with the touchscreen. At the time-point when the animal correctly touched the square on the touchscreen, we extracted a video frame of the XBI camera (Figure above, Video S1). We applied the following rules to select the nine correct interactions for each animal: 1) each session was divided in three periods: 15-30 min, 30-60 min and 60-90 min after start of the session. 2) In each time period, we picked the first three consecutive trials that were performed correctly by the animal. 3) If there were no three consecutive correct trials in a time period, then the second interaction of the first two consecutive correct interactions was chosen. We only applied this rule once (animal Alw, frame 6).

The video frames in supplemental figure S1 show that the monkeys operated the XBI in a consistent way already after less than two weeks after the first expose to the device. In all occasions the monkeys had their lips at the metal pin which delivered the reward. Except for three trials in monkey Toa (frames 2, 7, 9), the monkeys were sitting in front of the XBI. Note, that for the last day of animal Toa (frames 7, 8, 9) the reward tube was set on a different height. The changed behavior shows that a change of the reward pin position can affect the posture of the animals. Although the animal interface is symmetric, three out of the five animals only used their right hand. Animal Nor used the right hand 3 times and left hand 6 times. Animal Fla used the right hand 4 times and the left hand 5 times. For studies requiring information about the used hand arm-specific, ID-tagging will be required. Overall, despite the short accommodation phase of 10 sessions, the animals showed quite consistent behavior in front of the XBI with their heads in a defined position. This helps to achieve tasks with standardized visual stimuli. We expect that at higher task difficulties with more demanding visual stimuli the viewing of the screen will be even more consistent.
